# Prenatal Ambient Particulate Matter Exposure and Longitudinal Weight Growth Trajectories in Early Childhood

**DOI:** 10.3390/ijerph17041444

**Published:** 2020-02-24

**Authors:** Anna S. Rosofsky, M. Patricia Fabian, Stephanie Ettinger de Cuba, Megan Sandel, Sharon Coleman, Jonathan I. Levy, Brent A. Coull, Jaime E. Hart, Antonella Zanobetti

**Affiliations:** 1 Department of Environmental Health, School of Public Health, Boston University, Boston, MA 02118, USA; pfabian@bu.edu (M.P.F.); Megan.Sandel@bmc.org (M.S.); jonlevy@bu.edu (J.I.L.); 2 Department of Pediatrics, School of Medicine, Boston University, Boston, MA 02118, USA; sedc@bu.edu; 3 Biostatistics and Epidemiology Data Analytics Center, School of Public Health, Boston University, Boston, MA 02118, USA; sharcole2@gmail.com; 4 Department of Biostatistics, T.H. Chan School of Public Health, Harvard University, Boston, MA 02115, USA; bcoull@hsph.harvard.edu; 5 Department of Environmental Health, T.H. Chan School of Public Health, Harvard University, Boston, MA 02115, USA; azanobet@hsph.harvard.edu (A.Z.); rejch@channing.harvard.edu (J.E.H.); 6 Channing Division of Network Medicine, Department of Medicine, Brigham and Women’s Hospital and Harvard Medical School, Boston, MA 02115, USA

**Keywords:** air pollution, PM_2.5_, weight trajectories, in utero exposures, growth, childhood

## Abstract

Air pollution exposure during pregnancy has been associated with impaired fetal growth and postnatal weight gain, but few studies have examined the effect on weight growth trajectories. We examine the association between validated 1 km^2^ resolution particulate matter (PM_2.5_) concentrations, averaged over pregnancy, and sex-specific growth trajectories from birth to age six of participants in the Boston-based Children’s HealthWatch cohort (4797 participants, 84,283 measures). We compared weight trajectories, predicted using polynomial splines in mixed models, between prenatal PM_2.5_ above or below the median (9.5 µg/m^3^), and examined birth weight as an effect modifier. Females exposed to average prenatal PM_2.5_ ≥ 9.5 µg/m^3^ had higher weights compared to females exposed to < 9.5 µg/m^3^ throughout the study period (0.16 kg at 24 months, 0.61 kg at 60 months). In males, higher prenatal PM_2.5_ exposure was associated with significantly lower weights after 24 months of age, with differences increasing with time (−0.17 at 24 months, −0.72 kg at 60 months). Associations were more pronounced among low birth weight (<2500 g) females, but did not differ by birth weight status in males. Our findings demonstrate the complex association between air pollution exposures and childhood weight trajectories and emphasize the importance of sex-stratified analyses.

## 1. Introduction

Evidence is accumulating that weight growth trajectories in utero and during early postnatal periods are predictive of childhood overweight and obesity [1,2]. Investigating when the onset of childhood overweight and obesity occurs is of interest to understand the etiology of childhood and adult obesity and to identify critical periods for intervention [2]. Early-life overweight and obesity are associated with a range of chronic adverse health outcomes later in life, such as type 2 diabetes, coronary heart disease [3], and hypertension [4,5,6,7,8]. Although factors such as genetic susceptibility and nutrition are associated with overweight and obesity [9,10,11], the rapid rise of obesity implicates environmental risk factors as contributors to this trend [12]. Specific early-childhood growth trajectory phenotypes, such as the “thrifty phenotype” whereby catch-up growth follows fetal growth restriction, have been linked to adverse cardiometabolic outcomes in adulthood [13,14].

The vast majority of epidemiological studies to date associating prenatal ambient particulate matter with an aerodynamic diameter of 2.5 microns (PM_2.5_) and weight have focused almost exclusively on weight outcomes (birth weight, raw weight, body mass index (BMI), adiposity) measured cross-sectionally [15,16]. Modelling weight as a longitudinal outcome—growth trajectories—is a more informative measure than weight modelled as a cross-sectional outcome to understand steps on the causal pathway between early-life air pollution exposure and morbidities later in life. Few studies have investigated the link between prenatal ambient air pollution exposure and infant and early-childhood growth trajectories [17,18,19,20,21,22].

Using electronic health records (EHRs) and surveys administered to obtain detailed maternal and child demographic information, we investigated the association between prenatal PM_2.5_ exposure and weight growth trajectories from birth to age six years in the Boston-based Children’s HealthWatch (CHW) cohort. We assessed exposure using concentrations from spatially and temporally resolved PM_2.5_ predictions at 1 km^2^ resolution at maternal residence. Based on previous evidence associating prenatal PM_2.5_ and traffic exposure with low birth weight (LBW), we hypothesize a significant association between prenatal PM_2.5_ exposures and weight growth rates in our study population. 

## 2. Materials and Methods 

### 2.1. Study Population

We identified the study population from participants who enrolled in the CHW at the Boston Medical Center between 1 January 2008 and 31 December 2015. The CHW is an ongoing, sentinel surveillance study that gathers clinical and interview data from primary care sites or emergency department (ED) visits [23]. Institutional review board (IRB) approval (protocol number H-34069) was obtained from Boston University Medical Campus IRB prior to data collection and participants provided informed consent. 

At primary care or ED visits, trained CHW interviewers surveyed caregivers accompanying children younger than 48 months in a private setting. The survey covers multiple domains, including demographic and socioeconomic characteristics, breastfeeding practices, smoking status, child health status, and information about material hardship—including housing, food, and energy insecurity. Respondents were excluded if the interviewee was not the primary caregiver, if they did not speak English or Spanish, were not knowledgeable about the child’s household, had been interviewed previously that year, lived out of state, or did not consent to participate. Caregivers of critically ill or injured children were not approached. 

### 2.2. Linking EHR and CHW Survey Data

**Figure 1:**
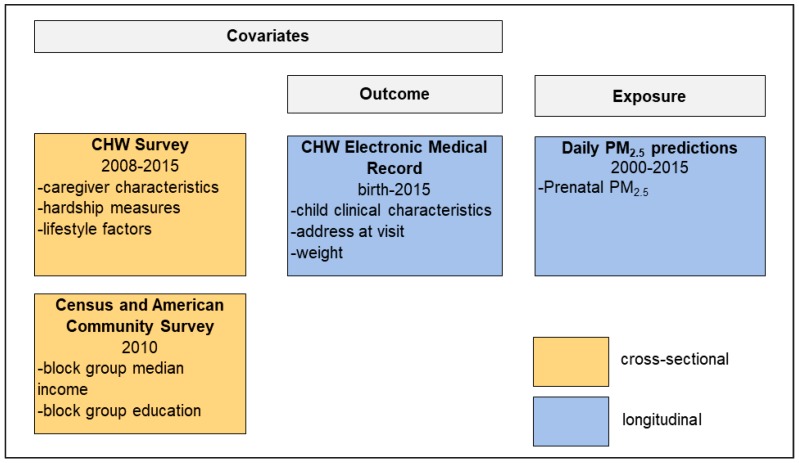
Data sources linked to create final analytical dataset for growth trajectory analysis. CHW: Children’s HealthWatch; PM_2.5_: particulate matter with an aerodynamic diameter of 2.5 microns.

As shown in Figure 1 and Figure S1, we linked CHW survey data to EHRs and to PM_2.5_ predictions. We matched CHW surveys to the EHR based on date of the child’s CHW interview, gender, date of birth, and medical record number. EHR data coverage included births until 31 December 2015, resulting in an EHR data range from 2005 to 2015. Birth weight and weight (kg) at each visit were extracted from the EHR, as well as address at each visit, age at each visit (months), gestational age (weeks), primary diagnosis for admission (ICD-10 code), child sex, child date of birth, and visit type (inpatient or outpatient). Missing EHR gestational age (42%) and birth weight (42%) data were imputed using CHW survey data. Correlation coefficients between the two data sources were 0.97 for birth weight and 0.95 for gestational age. We used the EHR value if CHW survey and EHR values differed.

### 2.3. Weight Outcome Data and Analytical Sample Selection

Of 100,712 visits, 84,283 visits included weight measurements. Only participants with two or more weight measurements over the study period were included in the study. We excluded visits with missing weight data, if exact weight measurements were repeated between visits, if an address was missing, could not be geocoded, or Boston Medical Center was listed as the address. Biologically implausible weight values, defined as a sex-specific weight-for-age z score of less than -6 or more than 5, were also dropped from analyses, as recommended by the Centers for Disease Control and Prevention [24]. This process yielded a final analytical sample of 4797 caregiver/child dyads with over 70,369 visits (Figure S1). 

In the final sample, 28%, 64%, and 8% of weight measurements were from ED, outpatient, and inpatient visits, respectively. We used weight measurements from all visit types as our dependent variable, and performed a sensitivity analysis excluding inpatient weights.

### 2.4. Exposure Assessment

*Geocoding.* We geocoded addresses recorded on the medical record to the corresponding residential parcel [25]. Of all addresses listed in the EHR, 0.8% were either missing or listed as a P.O. Box. Of the remaining addresses that were included in the geocoding process, 98% were matched to a residential address. We linked geocoded addresses at each visit to predicted PM_2.5_ data and several other spatial covariates, discussed in more detail below.

*Prenatal ambient PM_2.5_.* Details of the validated 1-km^2^ resolution PM_2.5_ prediction model can be found in Kloog et al. (2014). Briefly, this modeling approach used a combination of aerosol optical depth (AOD) satellite data retrieved using the multi-angle implementation of atmospheric correction (MAIAC) algorithm, land use and meteorological variables, and daily ambient monitor PM_2.5_ concentrations to calculate daily PM_2.5_ predictions on a 1-km^2^ grid between 2000 and 2015 [26]. The daily ambient PM_2.5_ monitoring data were obtained from the U.S. Environmental Protection Agency (EPA) Air Quality System (AQS) database, which includes ground monitoring sites located in urban, suburban, and rural areas across the United States, and from the Interagency Monitoring of Protected Visual Environments (IMPROVE) network, which includes monitors located in national parks and other federal lands.

We assigned PM_2.5_ to the geocoded addresses at birth using the closest 1 km^2^ grid centroid. We calculated average PM_2.5_ concentration over the prenatal period, which we estimated using date of birth and gestational age (in weeks). We categorized prenatal PM_2.5_ as a bivariate variable above and below the median (9.5 µg/m^3^) and as tertiles in a sensitivity analysis because of the non-linear association with weight. 

### 2.5. Covariates 

We assigned block group-level covariates from American Community Survey (ACS) 2006–2010 5-year summary data to each geocoded address. Linked covariates included median block group household income and percent with less than a high school degree (both continuous). 

Individual covariates collected from the EHR included number of moves within the study period (continuous), child’s birth weight (binomial categorized at > 2500 grams), child’s gestational age (continuous and binomial categorized at > 37 weeks), child sex, and birth date. 

We obtained the following covariates from the CHW survey: year of enrollment, caregiver BMI (underweight, normal weight, overweight, obese), caregiver race/ethnicity (non-Hispanic white, non-Hispanic black, Hispanic, other), breastfed during pregnancy (yes/no), caregiver smoking status in the past five years (yes/no), caregiver immigration status (U.S.-born, yes/no) and caregiver educational attainment (no schooling or some high school, high school, postsecondary), a composite measure of food, energy, and housing insecurity (referred to herein as “cumulative hardship”) [27], and mother’s age at birth (derived from child date of birth and mother’s age at CHW enrollment date).

### 2.6. Growth Trajectories Model

To estimate associations between average prenatal PM_2.5_ and growth trajectories, we applied a mixed effects model. We ran all models separately for males and females because of evidence of sex differences in growth trajectories and air pollution susceptibility [11,28,29]. In the model, we adjusted for several covariates a priori known to be biologically related to childhood postnatal weight, or as confounders of the association between weight and PM_2.5_. They include: caregiver race/ethnicity, cumulative hardship (categorical), child’s gestational age (categorical), block group median household income (continuous), and caregiver immigration status. Our main model also included a random intercept for child and a random slope for age to account for repeated measurements within subject and to adjust for heterogeneity in trends over time [30,31]; this model allows for correlated repeated weight measurements and varying number of measures per child. This modeling approach has been shown to produce good model fit in this and several other cohorts [9,32,33,34,35,36,37]. 

To model the growth trajectories we first applied generalized additive mixed effect models, controlling for covariates described above, and used penalized splines for age to visually inspect the shape of the relationship between weight and age defining sex-specific growth curves, and weight and PM_2.5_ [38]. 

To create a model that allows for a close approximation of the true growth function, we then used linear spline models with cubic polynomial terms for age. We used an iterative process to test combinations of one, two, three or four knot points at knot placements 3, 6, 9, 12, 18, 24, 36, and 40 months (number of participants with weight observations at different age ranges is shown in Supplemental Table S1). We explored two- and three- degree polynomial functions by adding linear, quadratic, and cubic terms to the model at the aforementioned knot points. We assessed model fit by comparing Akaike Information Criterion (AIC) and log-likelihood values between non-nested and nested models, respectively. Models for both sexes were the same and included a quadratic term, and cubic term, with knots at 6 and 12 months (Supplemental Table S2). We evaluated interactions between age terms and covariates using a likelihood ratio test. 

We found that including interactions between covariates and age terms in the model provided a significantly (*p*-value < 0.05) better fit than simply including the covariates as main effects, suggesting that the effect of covariates varies over age. We also tested year of enrollment to test for time trend, but found that it did not improve model fit. In addition, we included in the model interaction terms between prenatal PM_2.5_ and the age terms to allow the shape of the curve to differ between age groups. 

The final model was:

Y_ij_ = B_0_ + B_1_age_ij_ + B_2_age_ij_^2^ + B_3_age_ij_^3^ + B_4_(age_ij_ − 6months)^2^ + B_5_(age_ij_ − 12months)^2^ + B_6_exposure_i_ + B_7−n_covariates_i_ + (B_n_age_ij_*(exposure_i_ + covariates_i_)) + (B_n_age_ij_^2^*(exposure_i_ + covariates_i_)) + (B_n_age_ij_^3^*(exposure_i_ + covariates_i_)) + ((B_n_(age_ij_ − 6months)^2^)*(exposure_i_ + covariates_i_)) + (B_n_(age_ij_ − 12months)^2^*(exposure_i_ + covariates_i_)) + b_0i_ + b_1i_age_ij_ + e_ij,_ (1)

where Y_ij_ is weight for the i^th^ subject at time j, exposure_i_ is average prenatal PM_2.5_ for the i^th^ subject; covariates_i_ are non-time-varying covariates for the i^th^ subject, and b_0i_ and b_1i_ are the subject-specific random intercept and slope, respectively, for the i^th^ subject. 

To optimize interpretability of the model, we estimated predicted weight and associated 95% confidence interval at specified ages for the two levels of PM_2.5_. We then computed the differences in weight between levels of PM_2.5_ over the growth trajectory at the specified ages. 

We tested effect modification by birth weight (< 2500 grams), as growth trajectories may differ by birth weight. The phenotype of low birthweight followed by catchup growth has been associated with a range of cardiometabolic outcomes [13,14,39].

### 2.7. Sensitivity Analyses

We performed a sub-analysis including mother’s BMI (underweight, normal, overweight, obese) at study enrollment as a model covariate given literature showing that maternal and paternal weight are associated with childhood growth and obesity outcomes [9,10,11]. This analysis was conducted within a subset of the study population (63%) as the remaining participants were missing biological mother’s BMI. We also performed a sensitivity analysis excluding all subjects born <37 weeks gestation and a separate analysis excluding inpatient weights [40]. 

We report results as mean difference in weight (kg) between above and below median prenatal PM_2.5_ concentrations using *t*-tests, reported separately for males and females. All statistical tests were 2-tailed and a *p*-value of < 0.05 is used to denote statistical significance. Statistical analyses were conducted using R version 3.3 (R Foundation for Statistical Computing, Vienna, Austria).

## 3. Results

We present characteristics of the study population stratified by child sex in Table 1. The CHW cohort is an ethnically diverse, low-income population. Cohort participants were 50% non-Hispanic black and 35% Hispanic, 43% had a post-secondary degree, and 42% of mothers enrolled were immigrants to the United States. The average block group median household income was $43,792 and $43,442 (United States Dollar) for males and females respectively, which was lower than the state average of $70,114. The median mother’s age at delivery was 27 years (± 6.3). Average prenatal PM_2.5_ was approximately normally distributed across the population. The mean prenatal PM_2.5_ concentrations were similar between males (9.6 ± 1.2, range: 6.5–14.0 µg/m^3^) and females (9.5 ± 1.2, range: 6.3–14.1 µg/m^3^).

Supplemental Figure S2 displays the plot of the penalized spline model with a smooth term for model age in the model for weight, for males and females. As expected, both males and females have an exponential rate of growth in the first few months of life, which slows and becomes linear around 12 months of age, consistent with U.S. growth curve trends [41]. 

Supplemental Table S3 presents observed weights and estimated weights in the study population compared to the U.S. reference population weights from ages 0 to 72 months. Overall, models produced values close to observed weights. Cubic polynomial models slightly overestimated weight between 12 and 24 months for males, and slightly underestimated weights after 48 months for females. Observed weights in the study population were lower than the U.S. population during the early infancy period (0–3 months) but were higher at all other ages. 

**Figure 2:**
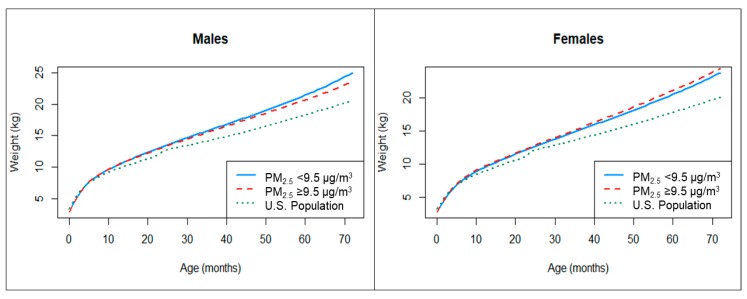
Predicted weight (kg) over age (months) by levels of average prenatal PM_2.5_. Note: Models adjusted for: age, age^2^, age^3^, quadratic spline terms at 6 and 12 months, gestational age, ethnicity, education, U.S.-born, cumulative hardship, and block group median income.

Figure 2 shows estimated childhood weight trajectories by levels of prenatal PM_2.5_ exposure below the median and above the median, and the weight trajectory of the general U.S. population for comparison, from birth through 72 months. Among males, above-median prenatal PM_2.5_ exposure results in growth trajectories that are significantly lower compared to the below-median prenatal PM_2.5_ exposure group from 2–6 years of age. The model predicts a 0.17 kg lower weight at 24 months and 0.72 kg lower weight at 60 months. We see an association in the opposite direction among females, where above-median prenatal PM_2.5_ is associated with significantly higher weights at all ages, with the exception of birth weight. The greatest difference in weight was at 72 months, where high exposure groups had weights 0.64 kg higher than low exposure groups (Table 2). 

In a sensitivity analysis, mother’s BMI at enrollment was associated with weight in all male prenatal PM_2.5_ models. Male babies of underweight mothers have statistically significantly lower weights compared to those from normal BMI mothers (−0.47, *p* = 0.0013), but not for female babies. The direction and magnitude of effects were similar when compared to a model that omitted BMI. We found similar results to our main analysis when we categorized PM_2.5_ into tertiles, with similar growth curves between the second and third tertiles of PM_2.5_ and lower rates of growth for those at or below the first tertile (data not shown, but available upon request). We restricted to full-term births (>37 weeks) and to outpatient weight measurements only, finding similar magnitude and direction of effect for male and female models (data not shown, but available upon request). 

**Figure 3:**
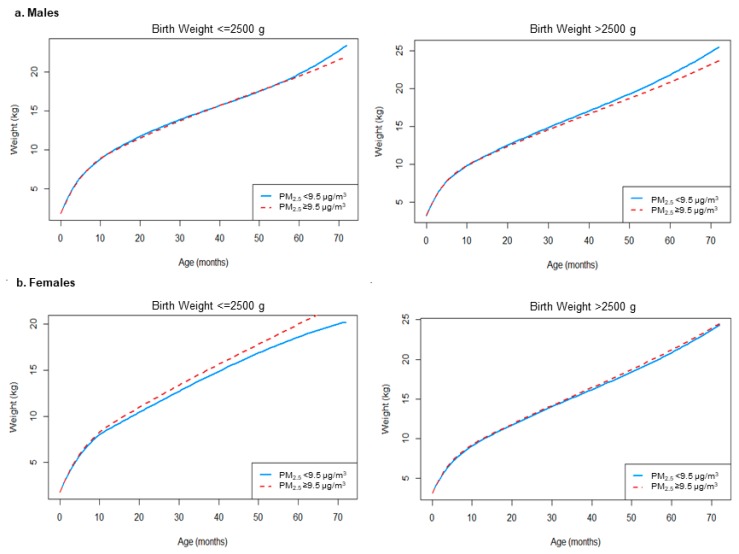
Predicted weight (kg) over age (months) by levels of average prenatal PM_2.5,_ stratified by birth weight categories for (**a**) males and (**b**) females. Note: Models adjusted for: age, age^2^, age^3^, quadratic spline terms at 6 and 12 months, gestational age, ethnicity, education, U.S.-born, cumulative risk, and block group median income

Results for polynomial age models for prenatal PM_2.5_, stratified by low birth weight (LBW, <2500 g) and non-low birth weight (>=2500 g), are presented in Figure 3, and Tables S4 and S5. As with the unstratified sample, estimated weights for males were significantly higher for the lower prenatal PM_2.5_ group in the non-LBW group, after 24 months. Absolute weight differences were more pronounced in the non-LBW group as compared to the LBW group (e.g., 0.82 kg in non-LBW vs. 0.39 kg in LBW at 60 months). Among females, differences between high and low PM_2.5_ exposure groups were greater among LBW compared to non-LBW females. Among LBW females, the above-median prenatal PM_2.5_ group had 0.24 kg greater weight at 6 months of age and 3.31 kg greater weight at 72 months compared to below-median PM_2.5_.

## 4. Discussion

We found differences in weight growth trajectories between levels of prenatal PM_2.5_ exposure in a racially and ethnically diverse population. Males exposed prenatally to PM_2.5_ greater than the median (9.5 µg/m^3^) had lower weights after 24 months of age compared to less-exposed groups. In contrast, over the growth trajectory (birth to age 6), female weights were greater for higher PM_2.5_ prenatal exposure, which was driven by the positive relationship between PM_2.5_ and weight in LBW females. 

Exposure to ambient air pollution such as particulate matter is a ubiquitous and modifiable risk factor. Inhalation of PM_2.5_ during pregnancy can interfere with fetal growth via oxidative stress (OS), intrauterine inflammation, endothelial function, and altered mitochondrial function [42,43,44]. These biological processes may alter trophic mechanisms that control growth throughout the life course [4]. 

The existence of sex-specific differences in the association between prenatal PM_2.5_ and growth trajectories is consistent with the broader literature on air pollution and birth outcomes, albeit with considerable variation in the magnitude and direction of effect. Overall, more studies have reported increased susceptibility to in utero PM_2.5_ exposure among males compared to females [28,45,46,47]. In a systematic review, females were more commonly found to be at higher risk of LBW, but in a re-analysis of data from four studies, males were at higher risk of LBW in the presence of high prenatal PM_2.5_ [45]. Ebisu and Bell (2012) reported a 3.2% (95% CI: 0.8, 5.6%) lower relative risk of LBW per IQR increase of PM_2.5_ elemental carbon in females compared to males [46]. The opposite effect was found in a pregnancy cohort located in Krakow: males had 188.6 g lower birth weight in the fourth compared to the first quartile of prenatal PM_2.5_ [47]. 

The literature is sparse with reference to prenatal outdoor ambient air pollution exposure and sex-specific differences in measures of weight later in childhood. Chiu et al. (2017) found that 1 µg/m^3^ increase in prenatal-average PM_2.5_ was associated with a 0.36 kg (95% CI: 0.12-0.68) increase in fat mass for males, but not females, and an increase in waist to hip ratio in females at four years of age [48]. Animal and human studies have demonstrated that prenatal PM_2.5_ exposure can induce sex-specific epigenetic modifications in leptin methylation, which is associated with adult metabolic disorders [49,50]. Sex-specific differences in energy metabolism and increased OS vulnerability in males have been found in animal studies, and may explain sex-specific differences found here [51]. 

Beyond the sex-specific effects, our findings are broadly consistent with a growing literature linking air pollution exposures with childhood growth [19,21,22,52]. In a Boston-area pregnancy cohort, Fleisch et al. (2015) found increased odds of weight-for-length > 95th percentile at 6 months of age in fourth quartile third-trimester PM_2.5_ and distance to roadway < 50 m compared to the referent groups, though estimates were not statistically significant. In a follow-up study, children whose mothers lived < 50 m from a major roadway at the time of delivery had 2.1 kg (95% CI: 0.8, 3.5) greater total fat mass compared to children (median 7.7 years of age) living >=200 m [52]. Inconsistent with our findings, this same study found each interquartile range increase in one-year average PM_2.5_ concentrations prior to each measurement occasion was associated with lower BMI-z score and total and truncal fat mass in mid-childhood (average 8 years of age) [52]. Investigators also examined trimester-specific associations, finding no association between third trimester PM_2.5_ and BMI outcomes [21]. 

A Massachusetts birth cohort with similar demographic characteristics to our study found an increased risk of overweight (BMI z-score >= 85^th^ percentile) and obesity (BMI z-score > = 95^th^ percentile) at ages 2–9 years in the highest versus lowest quartile of average prenatal PM_2.5_ exposure (OR = 1.3 (95% CI: 1.1, 1.6)) and postnatal PM_2.5_ in the first two years of life (OR = 1.2 (95% CI:1.1, 1.5)) [18]. Jerrett et al. (2014) assessed associations between traffic density within 150 m of the home and longitudinal sex-specific BMI growth trajectories between the ages of 5 and 11 years of age, finding no significant association [15]. 

The exposure and outcome assessments and study population sociodemographic characteristics may explain some of the discrepancies between our study and other studies that have assessed prenatal PM_2.5_ with weight trajectories. For instance, we used 1-km^2^ PM_2.5_ predictions, while Chiu et al. 2017 used measures from the nearest monitor, which may have decreased exposure variability and increased exposure measurement error. Other studies used BMI z-score and physiological measures of adiposity as their outcomes [18,19,20,48]. In the present study, we used raw-weights rather than z-scores to examine the true shape of the growth trajectory. Though McConnell et al. (2015) and Jerrett et al. (2014) found positive associations between postnatal near roadway pollution and traffic density with rates of BMI growth, their exposure metrics incorporated traffic density and meteorological conditions, averaged over the year of each measurement, whereas our study only considered PM_2.5_. 

Further, the CHW study population was more ethnically diverse and the prevalence of multiple hardships was higher as compared to the more ethnically homogenous and high-income Boston-area Project Viva cohort [19,21,52]. Inconsistencies may also be explained by our weaker measure of smoking (ascertained at the time of survey by asking whether caregiver smoked in the last 5 years), which is a predictor of weight. Greater cigarette smoke exposure and has been shown to have a synergistic effect with air pollution in increasing growth rates [20]. Further investigation is required to examine the role of other social determinants of health, such as housing conditions or food insecurity, acting independently or as modifiers of the association between ambient pollutants and weight gain. Using data from the CHW Boston site, we found that homelessness during pregnancy and average PM_2.5_ during the second trimester were marginally associated with reduced birthweight, while participating in Women, Infants, and Children (WIC) programs was associated with increased birthweight [53]. A deeper comprehension of these stressors can help identify root causes and potential solutions to the childhood obesity challenge, and why obesity may persist into adulthood [54]. 

There are a number of limitations in this study that may also explain our findings. We did not have measures of maternal smoking during pregnancy, nutrition, and maternal pre-pregnancy BMI, which are risk factors for childhood weight gain [9,55]. We attempted to control for these measures using a variable measuring smoking status during the past five years and maternal BMI at study enrollment. CHW does not collect information on diet, physical activity, and other environmental exposures that may be jointly associated with air pollution and weight gain, so there may be residual confounding in our analysis [56]. To address this concern, we controlled for both a measure of hardship, including food insecurity, and block group median income, both of which were associated with child growth in our models. 

There are some limitations inherent in the use of an EHR for data ascertainment. Health providers may inconsistently record weight measurements, resulting in non-differential outcome misclassification. However, weight measurements recorded in EHRs were found to be prone to < 0.7% error from 0 to 5 years of age according to a large prospective cohort study using EHRs [57]. Lastly, many of the visits in early childhood are routine checkups, whereas visits later in childhood may be comprised of children with poorer health outcomes, and thus differential susceptibility to the effects of PM_2.5_ exposure later in life. 

In spite of these limitations, our study has several strengths. This study included a large sample size: 4797 participants, with 70,369 weight measurements. The analytical method used to model the appropriate function of weight for age allowed us to explore longitudinal differences in trajectories by levels of ambient air pollution exposure. This method further allowed us take full advantage of the EHR containing measurements collected at varying time points and frequencies, and to estimate change in slope during specified growth time periods. Using an EHR for epidemiological analyses is a novel and relatively inexpensive, source of longitudinal data ascertainment, allowing for many measurements and limited potential for recall and participation bias [58]. The CHW cohort is ethnically diverse, making our results more representative of vulnerable populations, which are understudied to date. Survey data collected by CHW also provided rich information for covariates on multiple hardships, immigration status and caregiver characteristics. Up-to-date parcel-level reference data used in the geocoding process also strengthened confidence in exposure assessment. 

Our findings, and those of other studies examining early childhood weight trajectories, have multiple important public health implications. The associations in females are consistent with other risk factors implicated in the “thrifty phenotype” of low birthweight followed by rapid weight gain [2]. This phenotype has been linked to several morbidities in adulthood, including obesity, metabolic syndrome, type 2 diabetes, and cardiovascular disease [14]. Although the differences in weights between PM_2.5_ levels were small, the ubiquity of air pollution exposure across the population implies that even low levels of PM_2.5_ may shift population prevalence of excess weight gain over the life course. Differential weight trajectories, as noted here, have been shown to persist into late childhood and adulthood [11]. Our results point to a potentially susceptible period during which introduction of interventions known to promote healthy growth during the early childhood period could reduce the potential impacts of PM_2.5_ on growth [59].

## 5. Conclusions

In conclusion, we found in our study that low-birth weight females may be at increased susceptibility for weight gain in early childhood when exposed to higher prenatal PM_2.5_, with a significant inverse association among males. Studying growth trajectories, rather than attained measures of birth weight and BMI, provides an opportunity to understand susceptible phenotypes and periods of potential interventions. Because of the unique risk patterns found in the CHW population, additional studies are needed in a variety of different study populations and geographies to replicate our findings and to further explore the sex-specific differences found in this study. Future studies should also consider extending the follow-up period through adolescence and adulthood and the implications of specific growth trajectory phenotypes on adult morbidities, such as cardiovascular disease and diabetes. 

## Figures and Tables

**Table 1:** Characteristics of 4797 children and their caregivers enrolled in the Boston, Massachusetts based Children’s HealthWatch Cohort, 2008–2015.

Child Characteristics		
	Males (*n* (%))	Females (*n* (%))
Total	2603 (100)	2194 (100)
Birth weight (g) (% missing: 1.6 males, 1.1 females)		
<2500	292 (11.4)	289 (13.3)
>=2500	2269 (88.6)	1881 (86.7)
Gestational age (% missing: 0.4 males, 0.4 females)		
<37 weeks	411 (15.9)	309 (14.1)
>= 37 weeks	2181 (84.1)	1876 (85.9)
Breastfed During Pregnancy (% missing: 0.8 males, 0.6 females)		
Yes	1999 (77.4)	1688 (77.4)
No	584 (22.6)	492 (22.6)
Cumulative hardship (% missing: 12.7 males, 11.6 females) *^a^*		
0 hardships	806 (35.5)	655 (33.8)
1–3 hardships	1288 (56.7)	1121 (57.8)
>3 hardships	179 (7.9)	163 (8.4)
Number of overall visits (inpatient and outpatient)		
mean ± SD	14.8 ± 14.9	14.5 ± 14.1
Block group median income ($)		
mean ± SD	43,792.4 ± 22,003.7	43,442.3 ± 22,424.0
Self-reported caregiver characteristics		
Marital status (% missing: 0.5 males, 0.4 females)		
Married	933 (36.0)	782 (35.8)
Not married	1657 (64.0)	1403 (64.2)
Ethnicity (% missing: 1.2 males, 1.0 females)		
Hispanic	919 (35.7)	766 (35.3)
Black, non-Hispanic	1294 (50.3)	1099 (50.6)
White, non-Hispanic	222 (8.6)	191 (8.8)
Other	137 (5.3)	116 (5.3)
Education (% missing: 0.5 males, 0.3 females)		
Less than high school	611 (23.6)	524 (24.0)
High school graduate	853 (33.0)	723 (33.0)
Post-secondary	1125 (43.5)	941 (43.0)
Country of birth *^b^* (% missing: 0.8 males, 0.3 females)		
U.S.-born	1484 (57.5)	1266 (57.9)
Not U.S.-born	1099 (42.6)	921 (42.1)
Smoked in last 5 years (% missing: 4.1 males, 2.9 females)		
Yes	618 (24.8)	562 (26.4)
No	1879 (75.3)	1568 (73.6)
Age at child’s birth		
Mean ± SD	26.8 ± 6.3	27.0 ± 6.3

Note: SD, standard deviation; ^*a*^ Refers to biological mother; *^b^* Score derived from questions about housing, energy, and food insecurity.

**Table 2:** Mean predicted weight (kg) by prenatal PM_2.5_ category.

Prenatal PM_2.5_ Group	Birth	3 Months	6 Months	12 Months	18 Months	24 Months	36 Months	48 Months	60 Months	72 Months
Males (*n* = 2244; weight measurements = 32,405)										
<9.5 µg/m^3 *a*^	3.00	6.17	8.09	10.25	11.86	13.33	15.98	18.55	21.44	24.99
	(2.94, 3.06)	(6.11, 6.22)	(8.03, 8.15)	(10.17, 10.32)	(11.78, 11.96)	(13.22, 13.44)	(15.81, 16.14)	(18.34, 18.78)	(21.16, 21.72)	(24.62, 25.36)
≥9.5 µg/m^3 *a*^	3.02	6.17	8.14	10.29	11.77	13.16	15.71	18.16	20.72	23.60
	(2.95, 3.09)	(6.11, 6.23)	(8.07, 8.21)	(10.21, 10.37)	(11.68, 11.87)	(13.04, 13.28)	(15.54, 15.88)	(17.94, 18.38)	(20.44, 21.00)	(23.25, 23.94)
∆	−0.02	0.01	−0.05	−0.04	0.09	0.17	0.27	0.39	0.72	1.39
*p*-value ^*b*^	0.70	0.99	0.30	0.41	0.17	0.04	0.02	0.01	0.0003	<0.00001
Females (*n* = 1931; weight measurements = 27,148)										
<9.5 µg/m^3 *a*^	2.95	5.58	7.36	9.52	11.05	12.48	15.11	17.71	20.52	23.79
	(2.88, 3.02)	(5.52, 5.64)	(7.30, 7.42)	(9.45, 9.60)	(10.96, 11.15)	(12.35, 12.60)	(14.93, 15.30)	(17.45, 17.97)	(20.19, 20.83)	(23.35, 24.23)
≥9.5 µg/m^3 *a*^	2.98	5.71	7.55	9.70	11.21	12.64	15.41	18.18	21.13	24.42
	(2.91, 3.06)	(5.64, 5.78)	(7.48, 7.61)	(9.62, 9.79)	(11.11, 11.30)	(12.51, 12.76)	(15.22, 15.58)	(17.93, 18.41)	(20.83, 21.43)	(24.05, 24.81)
∆	−0.03	−0.13	−0.19	−0.19	−0.15	−0.16	−0.29	−0.47	−0.61	−0.64
*p*-value ^*b*^	0.50	0.006	0.0001	0.001	0.03	0.07	0.03	0.01	0.01	0.03

Note: All estimates are from polynomial mixed models adjusted for: age, age^2^, age^3^, quadratic spline terms at 6 and 12 months, gestational age, ethnicity, education, U.S.-born, cumulative risk and block group median income; ∆ = absolute difference in weight between low and high exposure categories (kg). *^a^* Values are mean estimated weights in kg (95% CIs); *^b^ p*-values for difference between low and high exposure categories.

## Supplementary Materials

The following are available online at https://www.mdpi.com/1660-4601/17/4/1444/s1. Table S1. Number of weight observations and subjects within each age range enrolled in the Boston, Massachusetts based Children’s HealthWatch Cohort, 2008–2015; Table S2. Estimates of Akaike Informaiton Criterion (AIC) obtained by mixed models to test optimal growth trajectory fit; Table S3. Observed and predicted weights (kg) in the study population compared to general U.S. population growth standards; Table S4. Estimated weight (kg) by PM_2.5_ categories, males, stratified by birth weight; Table S5. Estimated weight (kg) by PM_2.5_ categories, females, stratified by birth weight; Figure S1. Analytical sample selection from linked Children’s HealthWatch survey and electronic medical record data; Figure S2. Cubic regression splines illustrating weight (kg) growth by age generated from generalized additive models.

## References

[1] Baird J., Fisher D., Lucas P., Kleijnen J., Roberts H., Law C. (2005). Being big or growing fast: Systematic review of size and growth in infancy and later obesity. BMJ.

[2] Stettler N., Zemel B.S., Kumanyika S., Stallings V.A. (2002). Infant Weight Gain and Childhood Overweight Status in a Multicenter, Cohort Study. Pediatrics.

[3] Barker D.J.P., Osmond C., Forsén T.J., Kajantie E., Eriksson J.G. (2005). Trajectories of growth among children who have coronary events as adults. N. Engl. J. Med..

[4] Barker D., Eriksson J., Forsen T., Osmond C. (2002). Fetal origins of adult disease: Strength of effects and biological basis. Int. J. Epidemiol..

[5] Dennison B.A., Edmunds L.S., Stratton H.H., Pruzek R.M. (2006). Rapid infant weight gain predicts childhood overweight. Obesity (Silver Spring).

[6] Matthews E., Wei J., Cunningham S. (2017). Relationship between prenatal growth, postnatal growth and childhood obesity: A review. Eur. J. Clin. Nutr. Adv..

[7] Reilly J.J., Armstrong J., Dorosty A.R., Emmett P.M., Ness A., Rogers I., Steer C., Sherriff A. (2005). Early life risk factors for obesity in childhood: Cohort study. Br. Med. J..

[8] Afshin A., Forouzanfar M.H., Reitsma M.B., Sur P., Estep K., Lee A., Marczak L., Mokdad A.H., Moradi-Lakeh M., The GBD 2015 Obesity Collaborators Health (2017). Effects of Overweight and Obesity in 195 Countries over 25 Years. N. Engl. J. Med..

[9] Linabery A.M., Nahhas R.W., Johnson W., Choh A.C., Towne B., Odegaard A.O., Czerwinski S.A., Demerath E.W. (2013). Stronger influence of maternal than paternal obesity on infant and early childhood body mass index: The Fels Longitudinal Study. Pediatr. Obes..

[10] Parsons T.J., Power C., Manor O. (2001). Fetal and early life growth and body mass index from birth to early adulthood in 1958 British cohort: Longitudinal study. BMJ.

[11] Giles L.C., Whitrow M.J., Davies M.J., Davies C.E., Rumbold A.R., Moore V.M. (2015). Growth trajectories in early childhood, their relationship with antenatal and postnatal factors, and development of obesity by age 9 years: Results from an Australian birth cohort study. Int. J. Obes..

[12] Gillman M.W. (2005). Developmental Origins of Health and Disease. N. Engl. J. Med..

[13] Hales C.N., Barker D.J. (2001). The thrifty phenotype hypothesis. Br. Med. Bull..

[14] Vaag A.A., Grunnet L.G., Arora G.P., Brøns C. (2012). The thrifty phenotype hypothesis revisited. Diabetologia.

[15] Jerrett M., McConnell R., Wolch J., Chang R., Lam C., Dunton G., Gilliland F., Lurmann F., Islam T., Berhane K. (2014). Traffic-related air pollution and obesity formation in children: A longitudinal, multilevel analysis. Environ. Health.

[16] Zheng T., Zhang J., Sommer K., Bassig B.A., Zhang X., Braun J., Xu S., Boyle P., Zhang B., Shi K. (2016). Effects of environmental exposures on fetal and childhood growth trajectories. Ann. Glob. Health.

[17] Malmqvist E., Liew Z., Källén K., Rignell-Hydbom A., Rittner R., Rylander L., Ritz B. (2017). Fetal growth and air pollution -A study on ultrasound and birth measures. Environ. Res..

[18] Mao G., Nachman R.M., Sun Q., Zhang X., Koehler K., Chen Z., Hong X., Wang G., Caruso D., Zong G. (2016). Individual and Joint Effects of Early-Life Ambient PM2.5 Exposure and Maternal Pre-Pregnancy Obesity on Childhood Overweight or Obesity. Environ. Health Perspect..

[19] Fleisch A.F., Rifas-Shiman S.L., Koutrakis P., Schwartz J.D., Kloog I., Melly S.J., Coull B.A., Zanobetti A., Gillman M.W., Gold D.R. (2015). Prenatal Exposure to Traffic Pollution: Associations with Reduced Fetal Growth and Rapid Infant Weight Gain. Epidemiology.

[20] McConnell R., Shen E., Gilliland F.D., Jerrett M., Wolch J., Chih-Chieh C., Lurmann F., Berhane K. (2015). A Longitudinal Cohort Study of Body Mass Index and Childhood Exposure to Secondhand Tobacco Smoke and Air Pollution: The Southern California Children’s Health Study. Environ. Health Perspect..

[21] Fleisch A.F., Aris I.M., Rifas-Shiman S.L., Coull B.A., Luttmann-Gibson H., Koutrakis P., Schwartz J.D., Kloog I., Gold D.R., Oken E. (2019). Prenatal Exposure to Traffic Pollution and Childhood Body Mass Index Trajectory. Front. Endocrinol..

[22] Kim J.S., Alderete T.L., Chen Z., Lurmann F., Rappaport E., Habre R., Berhane K., Gilliland F.D. (2018). Longitudinal associations of in utero and early life near-roadway air pollution with trajectories of childhood body mass index. Environ. Health.

[23] Cutts D.B., Meyers A.F., Black M.M., Casey P.H., Chilton M., Cook J.T., Geppert J., De Cuba S.E., Heeren T., Coleman S. (2011). US housing insecurity and the health of very young children. Am. J. Public Health.

[24] Centers for Disease Control Growth Charts -WHO and CDC Child Growth Standards. https://www.cdc.gov/growthcharts/who_charts.htm#TheWHOGrowthCharts.

[25] MassGIS MassGIS Data -Master Address Data. http://www.mass.gov/anf/research-and-tech/it-serv-and-support/application-serv/office-of-geographic-information-massgis/datalayers/master-address-data.html.

[26] Kloog I., Chudnovsky A.A., Just A.C., Nordio F., Koutrakis P., Coull B.A., Lyapustin A., Wang Y., Schwartz J. (2014). A new hybrid spatio-temporal model for estimating daily multi-year PM2.5 concentrations across northeastern USA using high resolution aerosol optical depth data. Atmos. Environ..

[27] Frank D.A., Casey P.H., Black M.M., Rose-Jacobs R., Chilton M.M., Cutts D.B., March E., Heeren T., Coleman S., Ettinger de Cuba S. (2010). Cumulative Hardship and Wellness of Low-Income, Young Children: Multisite Surveillance Study. Pediatrics.

[28] Lakshmanan A., Chiu Y.H.M., Coull B.A., Just A.C., Maxwell S.L., Schwartz J., Gryparis A., Kloog I., Wright R.J., Wright R.O. (2015). Associations between prenatal traffic-related air pollution exposure and birth weight: Modification by sex and maternal pre-pregnancy body mass index. Environ. Res..

[29] Clougherty J.E. (2010). A growing role for gender analysis in air pollution epidemiology. Environ. Health Perspect..

[30] Fitzmaurice G.M., Laird N.M., Ware J.H. (2011). Applied Longitudinal Analysis.

[31] Howe L.D., Tilling K., Matijasevich A., Petherick E.S., Santos A.C., Fairley L., Wright J., Santos I.S., Barros A.J., Martin R.M. (2016). Linear spline multilevel models for summarising childhood growth trajectories: A guide to their application using examples from five birth cohorts. Stat. Methods Med. Res..

[32] Patel R., Tilling K., Lawlor D.A., Howe L.D., Bogdanovich N., Matush L., Nicoli E., Kramer M.S., Martin R.M. (2014). Socioeconomic differences in childhood length/height trajectories in a middle-income country: A cohort study. BMC Public Health.

[33] Tilling K., MacDonald-Wallis C., Lawlor D.A., Hughes R.A., Howe L.D. (2014). Modelling childhood growth using fractional polynomials and linear splines. Ann. Nutr. Metab..

[34] O’Keeffe L.M., Kearney P.M., Greene R.A., Zuccolo L., Tilling K., Lawlor D.A., Howe L.D. (2015). Maternal alcohol use during pregnancy and offspring trajectories of height and weight: A prospective cohort study. Drug Alcohol Depend..

[35] Chirwa E.D., Griffiths P.L., Maleta K., Norris S.A., Cameron N. (2014). Multi-level modelling of longitudinal child growth data from the Birth-to-Twenty Cohort: A comparison of growth models. Ann. Hum. Biol..

[36] Lourenço B.H., Villamor E., Augusto R.A., Cardoso M.A. (2012). Determinants of linear growth from infancy to school-aged years: A population-based follow-up study in urban Amazonian children. BMC Public Health.

[37] Grajeda L.M., Ivanescu A., Saito M., Crainiceanu C., Jaganath D., Gilman R.H., Crabtree J.E., Kelleher D., Cabrera L., Cama V. (2016). Modelling subject-specific childhood growth using linear mixed-effect models with cubic regression splines. Emerg. Themes Epidemiol..

[38] Wood S.N. (2017). Generalized Additive Models.

[39] UNICEF, WHO (2004). Low Birthweight: Country, Regional and Global Estimates.

[40] Reddy U.M., Bettegowda V.R., Dias T., Yamada-Kushnir T., Ko C.-W., Willinger M. (2011). Term pregnancy: A period of heterogeneous risk for infant mortality. Obstet. Gynecol..

[41] Ogden C.L., Kuczmarski R.J., Flegal K.M., Mei Z., Guo S., Wei R., Grummer-Strawn L.M., Curtin L.R., Roche A.F., Johnson C.L. (2002). Centers for Disease Control and Prevention 2000 growth charts for the United States: Improvements to the 1977 National Center for Health Statistics version. Pediatrics.

[42] Kannan S., Misra D.P., Dvonch J.T., Krishnakumar A. (2006). Exposures to airborne particulate matter and adverse perinatal outcomes: A biologically plausible mechanistic framework for exploring potential effect modification by nutrition responses to PM exposures. Environ. Health Perspect..

[43] Janssen B.G., Janssen B.G., Byun H., Gyselaers W., Lefebvre W., Andrea A. (2016). Placental mitochondrial methylation and exposure to airborne particulate matter in the early life environment: An ENVIRONAGE birth cohort study to airborne particulate matter in the early life. Epigenetics.

[44] de melo J.O., Soto S.F., Katayama I.A., Wenceslau C.F., Pires A.G., Veras M.M., Furukawa L.N.S., de Castro I., Saldiva P.H.N., Heimann J.C. (2015). Inhalation of fine particulate matter during pregnancy increased IL-4 cytokine levels in the fetal portion of the placenta. Toxicol. Lett..

[45] Ghosh R., Rankin J., Pless-Mulloli T., Glinianaia S. (2007). Does the effect of air pollution on pregnancy outcomes differ by gender? A systematic review. Environ. Res..

[46] Ebisu K., Bell M.L. (2012). Airborne PM2.5 chemical components and low birth weight in the northeastern and Mid-Atlantic regions of the United States. Environ. Health Perspect..

[47] Jedrychowski W., Perera F., Mrozek-Budzyn D., Mroz E., Flak E., Spengler J.D., Edwards S., Jacek R., Kaim I., Skolicki Z. (2009). Gender differences in fetal growth of newborns exposed prenatally to airborne fine particulate matter. Environ. Res..

[48] Chiu Y.H., Hsu H.H., Wilson A., Coull B.A., Pendo M.P., Baccarelli A., Kloog I., Schwartz J., Wright R.O., Taveras E.M. (2017). Prenatal particulate air pollution exposure and body composition in urban preschool children: Examining sensitive windows and sex-specific associations. Environ. Res..

[49] Chen M., Wang X., Hu Z., Zhou H., Xu Y., Qiu L., Qin X. (2017). Programming of mouse obesity by maternal exposure to concentrated ambient fine particles. Part. Fibre Toxicol..

[50] Calderón-Garcidueñas L., Torres-Jardón R., Kulesza R.J., Park S.-B., D’Angiulli A. (2014). Air pollution and detrimental effects on children’s brain. The need for a multidisciplinary approach to the issue complexity and challenges. Front. Hum. Neurosci..

[51] Mauvais-Jarvis F. (2015). Sex differences in metabolic homeostasis, diabetes, and obesity. Biol. Sex Differ..

[52] Fleisch A.F., Luttmann-Gibson H., Perng W., Rifas-Shiman S.L., Coull B.A., Kloog I., Koutrakis P., Schwartz J.D., Zanobetti A., Mantzoros C.S. (2016). Prenatal and early life exposure to traffic pollution and cardiometabolic health in childhood. Pediatr. Obes..

[53] Rhee J., Patricia Fabian M., de Cuba S.E., Coleman S., Sandel M., Lane K.J., Sade M.Y., Hart J.E., Schwartz J., Kloog I. (2019). Effects of maternal homelessness, supplemental nutrition programs, and prenatal PM2.5 on birthweight. Int. J. Environ. Res. Public Health.

[54] Cunningham S.A., Datar A., Narayan K.M.V., Kramer M.R. (2017). Entrenched obesity in childhood: Findings from a national cohort study. Ann. Epidemiol..

[55] Suzuki K., Sato M., Zheng W., Shinohara R., Yokomichi H., Yamagata Z. (2015). Childhood growth trajectories according to combinations of pregestational weight status and maternal smoking during pregnancy: A multilevel analysis. PLoS ONE.

[56] Jerrett M., McConnell R., Chang C.C.R., Wolch J., Reynolds K., Lurmann F., Gilliland F., Berhane K. (2010). Automobile Traffic around the Home and Attained Body Mass Index: A Longitudinal Cohort Study of Children aged 10–18 Years. Prev. Med..

[57] Smith N., Coleman K.J., Lawrence J.M., Quinn V.P., Getahun D., Reynolds K., Chen W., Porter A.H., Jacobsen S.J., Koebnick C. (2010). Body weight and height data in electronic medical records of children. Int. J. Pediatr. Obes..

[58] Casey J.A., Schwartz B.S., Stewart W.F., Adler N.E. (2016). Using Electronic Health Records for Population Health Research: A Review of Methods and Applications. Annu. Rev. Public Health.

[59] Braun K.V., Erler N.S., Kiefte-de Jong J.C., Jaddoe V.W.V., van den Hooven E.H., Franco O.H., Voortman T. (2016). Dietary Intake of Protein in Early Childhood Is Associated with Growth Trajectories between 1 and 9 Years of Age. J. Nutr..

