# Supplementary material for: Prenatal Ambient Particulate Matter Exposure and Longitudinal Weight Growth Trajectories in Early Childhood
Source: Int J Environ Res Public Health. 2020 Feb 24;17(4):1444. doi: 10.3390/ijerph17041444 (PMC7068568; doi:10.3390/ijerph17041444)
Supplement: Supplementary file 1 [file ijerph-17-01444-s001.pdf]

Supplemental Material

Prenatal Ambient Particulate Matter Exposure and Longitudinal Weight Growth Trajectories in Early Childhood

Anna Rosofsky, M. Patricia Fabian, Stephanie Ettinger de Cuba, Megan Sandel, Sharon Coleman, Jonathan I. Levy, Brent A. Coull, Jaime E. Hart, Antonella Zanobetti

Table S1. Number of weight observations and subjects within each age range enrolled in the Boston, Massachusetts based Children's HealthWatch Cohort, 2008-2015. 1
Table S2. Estimates of Akaike Informaiton Criterion (AIC) obtained by mixed models to test optimal growth trajectory fit. 2
Table S3. Observed and Predicted Weights (kg) in Study Population compared to General U.S. Population Growth Standards 3
Table S4. Estimated weight (kg) by $PM_{2.5}$ Categories, Males, Stratified by Birth Weight 6
Table S5. Estimated weight (kg) by $PM_{2.5}$ Categories, Females, Stratified by Birth Weight........... 7

Figure S1. Analytical sample selection from linked Children's HealthWatch survey and electronic medical record data
Figure S2. Cubic regression splines illustrating weight (kg) growth by age generated from generalized additive models.

Table S1. Number of weight observations and subjects within each age range enrolled in the Boston, Massachusetts based Children's HealthWatch Cohort, 2008-2015.

	Male		Female	
Age Range	n	measures	n	measures
0	1,667	1,667	1,441	1,441
0-3	631	891	550	751
3-6	818	1112	731	1025
6-12	732	989	645	865
12-18	577	740	429	609
18-24	512	641	395	529
24-36	325	398	264	317
36-48	217	265	177	197
48-60	155	133	90	107
60-72	87	72	61	68

Table S2. Estimates of Akaike Informaiton Criterion (AIC) obtained by mixed models to test optimal growth trajectory fit.

Knot placement months	cubic		cubic, quad knots	
	m	f	m	f
3	104744.8	88145.15	105794.8	88693.17
6	102438.3	86786.66	103326.2	87344.02
12	102821.4	86588.11	101981	86348.87
18	105806.2	88130.94	102806.9	86589.46
24	109315.3	90223.1	104206.8	87257.19
3, 6	102269.1	86630.48	102504.4	86833.27
3, 12	102174.5	86399.55	101982.9	86333.32
3, 18	103002	86898.68	102401.7	86485.47
3, 24	103972.4	87541.8	103013.3	86815.09
6, 12	101958.6	86328.46	101978.6	86335.82
6, 18	102054.5	86417.76	102095	86392.17
6, 24	102221.6	86557.97	102303.3	86526.5
12, 18	102536.9	86508.93	101982.8	86328.32
12, 24	102689.5	86565.07	101982.9	86325.06
18, 24	104541.7	87535.6	102319.2	86440.35

Table S3. Observed and Predicted Weights (kg) in Study Population compared to General U.S. Population Growth Standards

	Male			Female		
Age (months)	Observed[a]	Cubic model prediction	U.S. Population[b]	Observed[a]	Cubic model prediction	U.S. Population.[b]
0	3.2	3.1	3.4	3.1	3.0	3.2
3	6.1	6.2	6.4	5.5	5.6	5.8
6	8.0	8.1	7.9	7.3	7.5	7.3
12	10.0	10.3	9.6	9.5	9.6	8.9
18	11.7	11.8	10.9	11.1	11.1	10.2
24	13.1	13.3	12.2	12.5	12.5	11.5
36	15.8	15.8	14.3	15.1	15.2	13.8
48	18.5	18.4	16.3	18.1	17.9	15.9
60	21.3	21.0	18.5	20.9	20.8	18.0
72	23.3	24.1	20.5	24.9	24.1	20.3

[a]median observed weight from EMR within 2 weeks of listed age, unadjusted

[b]U.S. Population weights derived from WHO growth standards for ages 0-2 years, and CDC growth standards for ages 2-6 years

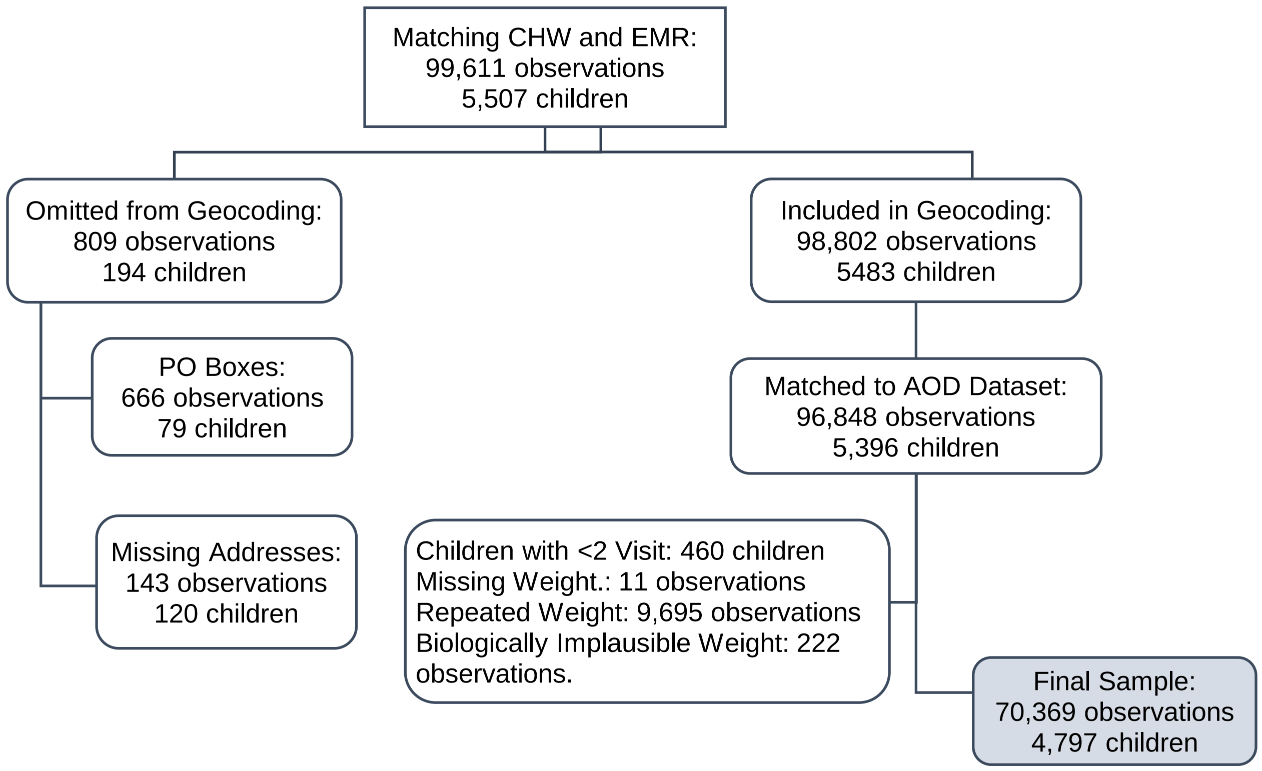

Figure S1. Analytical sample selection from linked Children's HealthWatch survey and electronic medical record data

Note: AOD, aerosol optical depth; CHW, Children's HealthWatch; EMR, Electronic Medical Record.

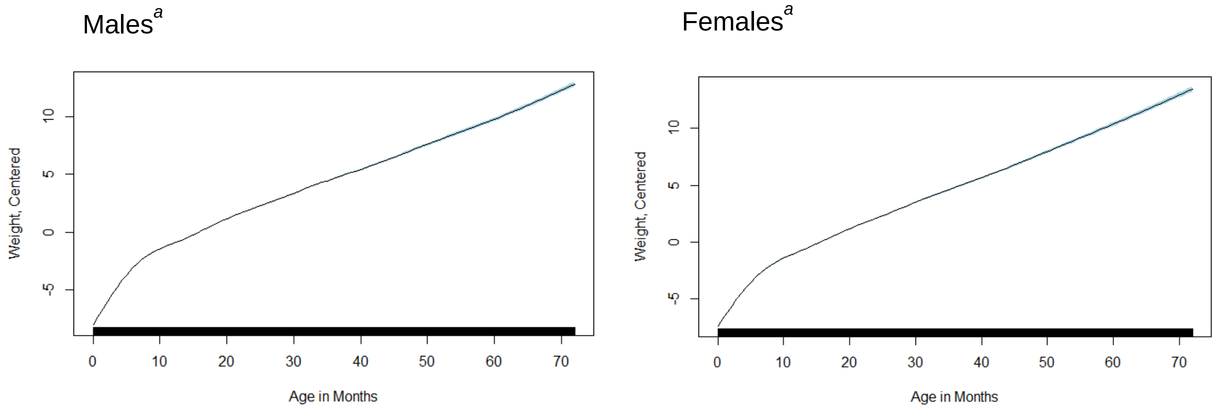

Figure S2. Cubic regression splines illustrating weight (kg) growth by age generated from generalized additive models.

Note: Adjusted for gestational age, cumulative hardship, median block group income, and ethnicity with 95% confidence intervals

[a]y-axis is centered at the mean; 95% confidence intervals (in light blue) are very small and are only visible towards the end of the growth curve

Table S4. Estimated weight (kg) by $PM_{2.5}$ Categories, Males, Stratified by Birth Weight

Prenatal $PM_{2.5}$ Group	Birth	3 months	6 month	12 months	18 months	24 months	36 months	48 months	60 months	72 months
Not Low Birth Weight - >2,500 g, (1,972 IDs, 27,027 measures)										
<9.5 μg/m^{3a}	3.20 (3.13, 3.26)	6.37 (6.31, 6.43)	8.27 (8.21, 8.33)	10.38 (10.30, 10.45)	11.98 (11.89, 12.08)	13.45 (13.33, 13.57)	16.13 (15.96, 16.31)	18.78 (18.54, 19.01)	21.73 (21.43, 22.03)	25.36 (24.95, 25.76)
≥ 9.5 μg/m^{3a}	3.21 (3.14, 3.28)	6.38 (6.31, 6.45)	8.32 (8.25, 8.39)	10.41 (10.33, 10.49)	11.88 (11.78, 11.98)	13.26 (13.13, 13.38)	15.83 (15.65, 16.01)	18.32 (18.08, 18.55)	20.91 (20.62, 21.21)	23.82 (23.44, 24.19)
Δ	-0.02	-0.01	-0.04	-0.03	0.11	0.19	0.31	0.46	0.82	1.54
p-value [b]	0.73	0.98	0.35	0.57	0.13	0.03	0.02	0.01	0.0001	< 0.00001
Low Birth Weight ≤2,500 g (245 IDs, 5,185 measures)										
<9.5 μg/m^{3a}	1.87 (1.67, 2.06)	5.02 (4.85, 5.19)	7.09 (6.92, 7.26)	9.52 (9.32, 9.72)	11.19 (10.94, 11.43)	12.62 (12.31, 12.93)	15.04 (14.59, 15.48)	17.3 (16.70, 17.90)	19.94 (19.17, 20.70)	23.47 (22.44, 24.49)
≥ 9.5 μg/m^{3a}	1.84 (1.62, 2.06)	4.89 (4.69, 5.09)	7.02 (6.82, 7.22)	9.50 (9.27, 9.72)	11.03 (10.76, 11.30)	12.42 (12.09, 12.75)	14.91 (14.45, 15.37)	17.2 (16.60, 17.81)	19.55 (18.79, 20.30)	22.19 (21.26, 23.11)
Δ	0.03	0.13	0.07	0.02	0.16	0.2	0.13	0.1	0.39	1.28
p-value [b]	0.84	0.34	0.61	0.88	0.40	0.40	0.70	0.82	0.48	0.07

Note: All estimates are from polynomial mixed models adjusted for: age, age^2, age^3, quadratic spline terms at 6 and 12 months, gestational age, ethnicity, education, U.S. born, cumulative risk and block group median income; Δ=absolute difference in weight between low and high exposure categories (kg)

[a]Values are mean estimated weights in kg (95% CIs)

[b]p-values for difference between low and high exposure categories

Table S5. Estimated weight (kg) by $PM_{2.5}$ Categories, Females, Stratified by Birth Weight

Prenatal $PM_{2.5}$ Group	Birth	3 months	6 months	12 months	18 months	24 months	36 months	48 months	60 months	72 months
Not Low Birth Weight>2500 g, (1,670 IDs, 22,928 measures)										
<9.5 μg/m^{3}[a]	3.15 (3.08, 3.21)	5.79 (5.72, 5.86)	7.55 (7.48, 7.62)	9.68 (9.60, 9.77)	11.24 (11.13, 11.35)	12.67 (12.53, 12.81)	15.30 (15.10, 15.50)	17.91 (17.64, 18.18)	20.84 (20.49, 21.19)	24.42 (23.94, 24.90)
≥ 9.5 μg/m^{3}[a]	3.16 (3.08, 3.23)	5.89 (5.82, 5.96)	7.70 (7.62, 7.77)	9.81 (9.73, 9.90)	11.32 (11.22, 11.43)	12.76 (12.62, 12.89)	15.49 (15.30, 15.68)	18.23 (17.97, 18.48)	21.18 (20.86, 21.50)	24.56 (24.16, 24.96)
Δ	-0.01	-0.10	-0.14	-0.13	-0.08	-0.09	-0.19	-0.32	-0.34	-0.14
p-value[b]	0.86	0.05	0.004	0.03	0.28	0.36	0.18	0.10	0.16	0.70
Low Birth Weight - ≤2500 g (247 IDs, 4,128 measures)										
<9.5 μg/m^{3}[a]	1.81 (1.62, 2.00)	4.45 (4.29, 4.61)	6.35 (6.19, 6.50)	8.60 (8.41, 8.79)	10.02 (9.78, 10.27)	11.40 (11.08, 11.72)	13.98 (13.49, 14.46)	16.28 (15.61, 16.95)	18.23 (17.36, 19.10)	19.79 (18.59, 20.98)
≥ 9.5 μg/m^{3}[a]	1.84 (1.61, 2.07)	4.55 (4.36, 4.74)	6.58 (6.40, 6.77)	8.95 (8.74, 9.16)	10.33 (10.06, 10.59)	11.78 (11.43, 12.12)	14.81 (14.29, 15.32)	17.83 (17.13, 18.53)	20.66 (19.77, 21.55)	23.09 (21.95, 24.24)
Δ	-0.03	-0.10	-0.24	-0.34	-0.30	-0.38	-0.83	-1.55	-2.43	-3.31
p-value[b]	0.84	0.42	0.05	0.02	0.10	0.11	0.02	0.002	0.0002	0.0001

Note: All estimates are from polynomial mixed models adjusted for: age, age^2, age^3, quadratic spline terms at 6 and 12 months, gestational age, ethnicity, education, U.S. born, cumulative risk and block group median income; Δ=absolute difference in weight between low and high exposure categories (kg)

[a]Values are mean estimated weights in kg (95% CIs)

[b]p-values for difference between low and high exposure categories
